# Rapid Turnaround Time for Toxic Alcohol Testing Is Associated with Shorter Hospital Length of Stay

**DOI:** 10.5811/westjem.56974

**Published:** 2026-07-16

**Authors:** Steven Chen, Jacob Yudiono, Varun Vohra, Rebecca Lipscomb, Rahul Mhaskar, Justin K. Arnold, Diana Dean

**Affiliations:** * USF Morsani College of Medicine, Tampa, Florida; † Wayne State University School of Medicine, Department of Emergency Medicine, Detroit, Michigan; ‡ Wayne State University School of Medicine, Michigan Poison & Drug Information Center, Detroit, Michigan; § USF Morsani College of Medicine, Department of Medical Education, Tampa, Florida; ¶ Florida Poison Information Center – Tampa, Tampa, Florida; || USF Morsani College of Medicine, Department of Internal Medicine, Tampa, Florida; # University of South Florida, Department of Emergency Medicine, Tampa, Florida

## Abstract

**Introduction:**

Toxic alcohol poisoning (methanol or ethylene glycol) can cause severe metabolic disturbances, end-organ damage, and death. Timely diagnosis is paramount and predicated on history, lab markers, and confirmatory laboratory testing, which inform antidote use and/or hemodialysis. Confirmatory toxic alcohol testing capabilities and turnaround times vary by geographic region. Our objective in this study was to evaluate how differences in toxic alcohol testing turnaround times impact our primary outcome of hospital length of stay and secondary outcomes of antidote (fomepizole) administration and medical outcomes (including death, major, moderate, and minor effects) among patients with suspected toxic alcohol poisoning.

**Methods:**

This is a retrospective cohort study of toxic alcohol poisoning cases reported to two U.S. poison centers, Michigan Poison and Drug Information Center (MiPDC) and Florida’s Poison Information Network (FPIN), from 2018–2022. Based on investigator experience with each center’s practice patterns, MiPDC had a toxic alcohol testing turnaround time of 12–24 hours, while FPIN had turnaround times averaging 5–7 days. We queried poison center databases for cases where fomepizole was recommended and/or administered. Medical outcomes were categorized using National Poison Database System definitions: minor effects (minimally bothersome symptoms); moderate effects (pronounced symptoms requiring treatment); major effects (life-threatening or significant disability); and death. We excluded cases if there was no concern for toxic alcohol poisoning or if fomepizole was administered for acetaminophen poisoning.

**Results:**

A total of 963 cases met inclusion criteria: (MiPDC, *n* = 590; and FPIN, *n* = 373). Patients reported to FPIN had a longer median hospital length of stay compared to MiPDC (65.0 versus 48.0 hours, *P* < .001). FPIN patients received more doses of fomepizole (2.0 versus 1.0 doses, *P* < .001). FPIN patients had 4.1 times the odds of major effect (95% CI, 1.3–13.3) (*P* = .02) and 4.7 times the odds of minor effect (95% CI, 1.4–15.8) (*P* = .01), without significant association for moderate effects or death.

**Conclusion:**

Access to rapid confirmatory toxic alcohol testing was associated with shorter hospital length of stay, reduced antidote use, and improved medical outcomes in suspected toxic alcohol poisonings. Future studies are warranted to comprehensively characterize the variation in region-specific care and inform efforts to standardize confirmatory testing capabilities.

## INTRODUCTION

Toxic alcohol poisoning in the United States is most associated with intentional or unintentional ingestions of substances containing methanol or ethylene glycol. In the absence of a confirmed or witnessed ingestion, toxic alcohol poisoning remains a clinical concern in patients presenting with high anion-gap metabolic acidosis. Clinical diagnosis is often predicated upon a combination of patient history, physical examination, laboratory results, and confirmatory toxic alcohol testing. Delays in diagnosis and treatment can lead to severe metabolic acidosis, permanent end-organ damage, and death. Thus, timely quantitative results and therapeutic interventions are critical.^1^

Quantitative toxic alcohol testing using gas or liquid chromatography with or without mass spectrometry (LC-MS) and/or flame ionization offers a highly sensitive and specific tool that can guide antidote administration with intravenous fomepizole and inform definitive treatment with hemodialysis.^1^ Healthcare facilities with confirmatory toxic alcohol testing capabilities can provide rapid results. Swift turnaround times aid clinicians with accurate and timely diagnoses and allow rapid de-escalation and discontinuation of hospital resources (eg, antidotes and/or hemodialysis) in the setting of negative toxic alcohol confirmatory testing.^2^

Readily available toxic alcohol measurements, however, are not routinely available in many clinical settings, with accessibility varying regionally. Clinicians without access to rapid confirmatory testing must evaluate possible toxic alcohol exposures using surrogate lab markers such as serial electrolytes and serum osmolality to gauge acid-base status, anion and osmolal gap calculations, and evolving end-organ damage. However, despite these lab markers being readily available, their use is associated with limitations, including interindividual variability of baseline values with the osmolal gap, the need for precise simultaneous lab draws, and potential confounding from other conditions that can also elevate the osmolal gap.^2^–^7^

In the setting of an unknown toxic alcohol concentration, clinicians often treat empirically with fomepizole (4-methylpyrazole, or 4-MP) with treatment regimens on the order of days being common.^8^ The literature is sparse regarding appropriate triage, treatment, and medical clearance guidance for toxic alcohol poisoned patients without confirmatory results.^2^ More routine and widespread use of testing capabilities has been limited at an institutional level, which in part may be related to its significant upfront (instrument and peripherals) and upkeep costs (maintenance, calibration, technical expertise).^2^,^9^ Nonetheless, readily available confirmatory lab testing may improve patient outcomes, optimize hospital resource allocation and use, and reduce healthcare expenditures.

In this study, we aimed to evaluate the impact of timely access to confirmatory toxic alcohol testing on patient care including therapeutic interventions, hospital length of stay (LOS), and medical outcomes. We hypothesized that access to rapid toxic alcohol testing—defined as access to confirmatory testing results within 24 hours—would be associated with decreased hospital LOS, less fomepizole administration, and improved medical outcomes.

**Population Health Research Capsule**

What do we already know about this issue?

*Toxic alcohol poisoning requires rapid diagnosis, but confirmatory testing availability and turnaround times vary across regions*.

What was the research question?

*Do toxic alcohol testing turnaround times affect length of stay, antidote use, and medical outcomes?*

What was the major finding of the study?

*Prolonged testing turnaround time was associated with a longer hospital length of stay (65 vs 48 hours, P < .001)*.

How does this improve population health?

*Expanding rapid testing may reduce hospital stays, limit unnecessary antidote use, and improve outcomes in suspected toxic alcohol poisoning*.

## METHODS

### Study Settings

This was a retrospective cohort study of suspected toxic alcohol cases, in which fomepizole was recommended/given and reported to two U.S. poison centers in different geographic regions with differing access to confirmatory toxic alcohol testing. The MiPDC covers a population of approximately 10 million people, receives between 55,000–60,000 calls per year, and covers approximately 200 inpatient healthcare facilities. The MiPDC coordinates toxic alcohol testing for much of its state by facilitating the transport of lab samples from outlying hospitals to have chromatographic testing performed at a hospital lab. Based on investigator experience, this results in a turnaround time of 12–24 hours in most cases. In contrast, all hospitals within the FPIN catchment area rely on third-party commercial labs for confirmatory toxic alcohol testing. Based on investigator experience, this results in turnaround times averaging 5–7 days. The FPIN covers a population of approximately 21 million people, receives about 113,000 calls per year, and covers approximately 360 inpatient healthcare facilities.

### Data Abstraction

We queried the two centers’ respective databases using ToxSentry (an electronic health record system for poison centers) for all toxic alcohol exposure cases between January 1, 2018–December 31, 2022, where fomepizole was recommended and/or administered (inclusion criteria). Cases were excluded if toxic alcohol poisoning was not mentioned in any part of the reviewed case narratives, if fomepizole was being administered for acetaminophen poisoning, and if there was a non-human exposure.

All extracted cases were de-identified. We recorded age, sex, reason (eg, intentional [self-harm, desire for intoxication], unintentional [exploratory, no intent of self-harm]), clinical effects, therapies, and medical outcome. Case narratives were reviewed for hospital LOS, time until first fomepizole dose, doses of fomepizole administered, historical features of presentation, details about hemodialysis session(s), and any documented quantitative toxic alcohol concentrations. Our primary outcome was hospital LOS, which we defined as the time of hospital entry to time of hospital discharge or time of last poison center follow-up. Secondary outcomes included the number of doses of fomepizole administered and patient outcomes.

We defined fomepizole dosage as the number of discrete doses (rather than weight-dependent vials) administered during a patient’s hospital stay. Typically, the loading dose of fomepizole is 15 milligrams per kilogram (mg/kg), with maintenance doses of 10mg/kg administered as needed thereafter. Detailed methods for abstraction of hospital LOS and doses of fomepizole administered are described in Appendices A and B. For subgroup analysis, patients were deemed to have a “known toxic alcohol ingestion in their presentation” when ingestion of a toxic alcohol was self-reported by the patient or witnessed by another person and subsequently reported to the clinician.

We coded medical outcomes (eg, major effects, moderate effects, minor effects, no effects, death) according to the predefined criteria set forth by the *National Poison Database System Coding Manual (NPDS)*.^10^ Cached under the banner of “minor effects” are cases that were not followed or could not be followed to a known outcome but were judged as likely nontoxic exposures, exposures with minimal clinical effects, or exposures deemed not responsible for the effects. We excluded cases that could not be followed but were judged as a potentially toxic exposure from statistical analyses comparing medical outcomes.

Chart abstraction was performed by two trained investigators (SC and JY) blinded to each other’s abstraction. A third study investigator (DD) reviewed cases with significant discrepancies between the two abstractions. Our study was compliant with the following medical record review criteria listed: abstractor training; case selection criteria; variable definition; abstraction forms; abstractor performance monitoring; interrater reliability discussion; interrater reliability testing; medical record identification; sampling method; and institutional review board approval.^11^

### Data Analysis

We used the Cohen kappa and the Spearman correlation coefficient to measure the agreement between chart abstractions. The Mann-Whitney U test compared hospital LOS between poison centers, sexes, and hemodialysis administration. We performed pairwise Spearman correlation coefficient analyses to measure the correlation between LOS, age, and doses of fomepizole. Pearson chi-squared test compared medical outcomes between poison centers and sex of patient. The Kruskal-Wallis test was performed to compare medical outcomes with age, time to first dose of fomepizole, and number of doses of fomepizole. We used the Hodges-Lehmann estimate to calculate median differences and confidence intervals for hospital LOS, doses of fomepizole, and time until first fomepizole dose between poison centers.

Linear regression and multinomial logistic regression were used to investigate the relationships between LOS (primary outcome) and medical outcomes (secondary outcome), respectively, adjusting for confounders (including age, sex, doses of fomepizole, time until first fomepizole dose, intent of overdose, and rates of increased anion gap). The LOS was logarithmically transformed to meet regression assumptions, and reported beta values have been exponentiated. One outlier from FPIN was removed from LOS analyses (725 hours).

**Figure 1:**
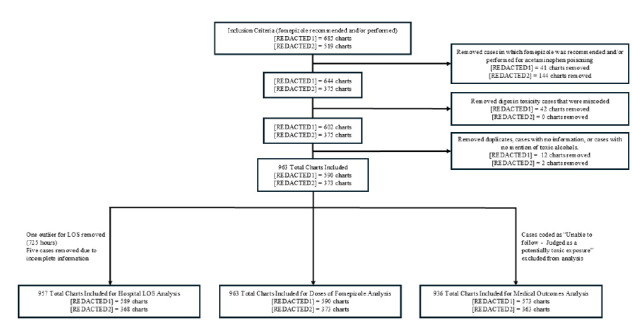
Study flow diagram in a study comparing the effect of turnaround times for testing between two U.S. poison centers, MiPDC and FPIN, in cases of suspected toxic alcohol exposures. *MiPDC*, Michigan Poison and Drug Information Center; *FPIN*, Florida’s Poison Information Network.

We conducted analysis using IBM SPSS v29 software (International Business Machines Corp, Armonokm NY), *P* values < .05 (alpha) were considered statistically significant. Figure 1 displays a study flow diagram. This study was reviewed and considered exempt by the institutional review board.

## RESULTS

Over the five-year study timeframe, 373 suspected toxic alcohol exposures were reported to the FPIN network, while 590 exposures were reported to MiPDC. The median ages were 43.0 (interquartile range [IQR], 28.0–56.0) at MiPDC and 47.0 (IQR, 35.0–58.0) at FPIN. There were 71 reported pediatric exposures (MiPDC, *n*. = 62/590 (10.5%) versus FPIN, *n* = 9/373 (2.4%). Major medical outcomes were most frequent at (MiPDC, *n* = 221/573 (38.6%) versus FPIN, *n* = 166/363 (45.7%). Acidosis, increased anion gap, and central nervous system depression were the most reported clinical findings. There were 71 reported deaths: (MiPDC, *n* = 39/575 (6.8%) versus FPIN, *n* = 32/363 (8.8%). Rates of dialysis, clinical effects, critical therapies, and intents of overdose from the two poison centers can be found in Table 1.

**Figure 2:**
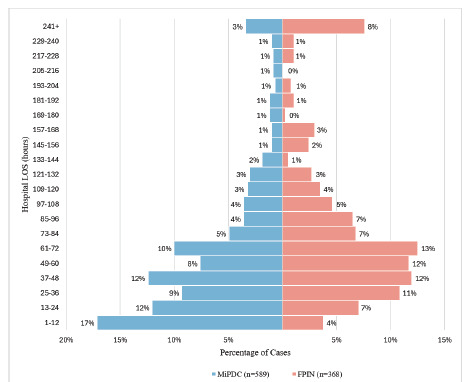
Distribution of hospital lengths of stay in a study comparing the effect of turnaround times in suspected toxic alcohol exposures between two U.S. poison centers. *LOS*, length of stay.

Comparative analyses revealed the median hospital LOS was longer in FPIN (65.0 hours [41.0–101.0] versus 48.0 hours [20.0–89.0]; *P* < .001) with a median difference of 18.0 hours (95% CI, 12.0–23.0). The median number of doses of fomepizole administered was higher in FPIN (2.0 [1.0–4.0] versus 1.0 [1.0–2.0]; *P* < .001), with a median difference of 0 doses (95% CI, 0.0–1.0). The median time (hours) to administration of the first dose of fomepizole was higher in FPIN (4.0 hours [2.5–8.5] versus 3.0 [1.5–5.0]; *P* < .001), with a median difference of 1.0 hours (95% CI, 0.5–2.0). Patients in FPIN were more likely to have at least one dose of fomepizole (92.8% versus 83.9%; *P* < .001). FPIN patients were more likely to have metabolic acidosis (62.7% vs. 51.9%; *P* < .001). Figure 2 illustrates the distribution of hospital LOS between MiPDC and FPIN.

There was a strong positive correlation between both abstractors’ datasets (*P* = .998) for both primary and secondary outcomes.

Upon bivariate linear regression analysis, hospital LOS was 19.8 hours shorter in the MiPDC group (95% CI, −30.3 to −9.3; *P* < .001). Adjusting for covariates, including age, sex, doses of fomepizole, time until first fomepizole dose, rates of increased anion gap, and intent, on multivariate analysis demonstrated that LOS in the FPIN group was higher by a factor of 1.2 (95% CI, 1.0–1.4) (*P* = .02). For every additional dose of fomepizole, the estimated LOS increased by a factor of 1.2 (95% CI, 1.2–1.2; *P* < .001). The effect of each variable on hospital LOS is listed in Table 2.

Bivariate analysis of medical outcomes indicated that FPIN had 4.7, 4.1, and 3.8 times the odds of major, moderate, and minor clinical effects, respectively (*P* < .001). On multivariate analysis, after adjusting for age, sex, doses of fomepizole, time until first fomepizole dose, and intent, FPIN had 4.1 times the odds of major effect (95% CI, 1.3–13.3) (*P* = .02) and 4.7 times the odds of minor effect (95% CI, 1.4–15.8) (*P* = .01). There was no significant association in the odds of moderate effect (95% CI, 1.0–10.2; *P* = .05) or death between groups (95% CI, 1.0–21.8; *P* = .06). Table 3 displays the association between various variables and patient medical outcomes.

In MiPDC, 68.1% of charts (*n* = 402/590) had a documented quantitative methanol concentration and 71.0% (*n* = 419/590) had a quantitative ethylene glycol concentration. In FPIN, 3.2% of charts (*n* = 12/373) had a documented quantitative methanol concentration and 7.0% (*n* = 26/373) had a quantitative ethylene glycol concentration. Table 4 displays data on the quantitative toxic alcohol concentration results obtained at MiPDC and FPIN.

### Subgroup Analysis

Among patients with a self-reported or witnessed (ie, “known”) toxic alcohol ingestion in their presentation (*n =* 447), the median LOS (hours) was higher in FPIN (54.0 hours [31.0, 82.0] versus 32.0 [9.0, 64.0]; *P* < .001). In this subgroup, the median number of fomepizole doses administered was higher in FPIN (2.0 hours [1.0, 4.0] versus 1.0 [1.0, 3.0]) (*P* = .003). In the subgroup of patients without a “known” toxic alcohol ingestion in their presentation (*n* = 516), FPIN exhibited a greater mean LOS (hours) (74.0 [50.0, 129.0] versus 63.0 [36.0, 118.0]) (*P* = .006). Median doses of fomepizole were not significantly different: 1.0 [1.0, 3.0] versus 1.0 [1.0, 2.0]) (*P* < .001).

## DISCUSSION

Our retrospective study highlights the importance of readily available access to rapid confirmatory testing in the management of patients with suspected toxic alcohol exposures. For our primary outcome, access to prompt confirmatory toxic alcohol testing capabilities in the MiPDC cohort was associated with a shorter median hospital LOS by 18 hours. Similarly, patients in the MiPDC group received fewer median doses of fomepizole. Our findings suggest that rapid confirmatory testing capabilities may enable clinicians to more quickly and accurately identify patients who require treatment with fomepizole and inform more appropriate discontinuation criteria. In contrast, lack of time-sensitive confirmatory testing requires ongoing clinician monitoring of surrogate laboratory markers, which are less sensitive and result in more extensive prophylactic treatment with antidote, prolonged hospitalizations, and increased healthcare resource utilization.

Toxic alcohol exposures reported to poison centers can largely be grouped based on whether there was a self-reported or witnessed ingestion of a toxic alcohol, or undifferentiated severe metabolic acidosis. Our data demonstrate that the use of healthcare resources (ie, LOS, fomepizole administration) was higher in the FPIN group. In patients who self-reported a toxic alcohol ingestion or had a witnessed ingestion, patient care is centered around determining whether the patient ingested sufficient quantities requiring fomepizole and/or hemodialysis.^1^ This information could be gleaned most accurately via quantitative toxic alcohol concentrations. We observed that in this subgroup of patients, the FPIN group, which lacked prompt confirmatory testing, was associated with a hospital LOS over 20 hours longer but only administered one additional dose of fomepizole. This relatively smaller difference in the number of fomepizole doses may be in part due to MiPDC’s practice of recommending a prophylactic dose of fomepizole while awaiting confirmatory quantitative testing results, while FPIN typically relies on trending serial metabolic panels.

For patients with no “known” history of toxic alcohol ingestion, suspicion of a toxic alcohol exposure is considered in patients presenting with a high anion gap metabolic acidosis.^1^ Our data show that patients in the FPIN group were associated with a longer hospital LOS of approximately 10 hours, potentially reflecting the inability to swiftly rule out toxic alcohol poisoning as a diagnosis without ready access to quantitative testing.

Prior research indicates that clinicians may be overtreating toxic alcohol poisoning with empiric fomepizole for patients who lack sufficient quantitative toxic alcohol concentrations to meet treatment threshold.^2^,^6^,^12^ Our findings show that across both populations, only 6.5% of methanol concentrations and 18.2% of ethylene glycol concentrations reached the standard threshold for continuous treatment with fomepizole (> 20 mg/dL). However, without ready access to confirmatory toxic alcohol testing, clinicians must rely on less-sensitive surrogate lab markers, which have implicit limitations (eg, osmolal gap) that may lead to clinician hesitancy with treatment discontinuation.^7^

Patients in the FPIN group had higher rates of acidosis, which may help explain the more significant medical outcomes. Patients in the FPIN group experienced a higher rate of major and minor clinical effects, although no difference in moderate clinical effects or mortality was observed. Whether this was due to a later presentation to the healthcare facility, late involvement of the managing poison center, more accurate charting or another factor, remains unclear. One potential explanation is that access to rapid LC-MS testing enables more definitive toxic alcohol treatment protocols that allow clinicians to either quickly identify a poisoned patient or consider other etiologies.

The ability to rapidly diagnose toxic alcohol poisoning also may have significant cost-savings implications. Between 2010–2021, over 24,000 cases of fomepizole administration for possible toxic alcohol exposure occurred in the United States.^13^ The cost for a vial of fomepizole (1.5 grams/1.5 milliliters) is $1,200–$2,000.^13^ With the limiting assumption of one vial per dose of fomepizole, the average patient from FPIN receiving a mean of 0.7 additional doses over MiPDC would incur an extra cost of $1,300. Expanding confirmatory testing capabilities represents a cost-effective investment that reduces unnecessary fomepizole doses, hospital LOS, and overall healthcare expenditures associated with resource use. Following conservative estimates of hospitalization cost of $1,937 per inpatient day, MiPDC, which had 590 suspected toxic alcohol exposures, saved an estimated total of $850,000 over the five-year study period.^14^

Like the region managed by FPIN, many areas in the United States lack ready access to rapid toxic alcohol testing. In 2022, a survey involving 36 U.S. poison centers concluded that only 19.4% (*n* = 7/36) reported regional healthcare facilities capable of confirmatory toxic alcohol testing results within 24 hours.^2^ Moreover, 61% of poison centers (*n* = 22/36) reported ≤ 10% of regional hospitals in their catchment area had access to 24-hour confirmatory testing facilities.^2^ Given these gaps, access to confirmatory LC-MS testing could have a widespread impact that should prompt further prospective and higher. powered retrospective studies to validate the effect of rapid toxic alcohol testing on toxic alcohol management. In today’s world of complex healthcare administrative decisions, our findings may generate further discussion and action for expanded confirmatory testing capabilities that can optimize both patient care and mitigate strains on resource demand in an already overburdened healthcare landscape.

## LIMITATIONS

This study had several limitations. Our dataset may not be a complete representation of all cases of possible toxic alcohol exposures in a region because poison control data rely on passive and voluntary information communicated by healthcare clinicians or callers from the public. Data may be skewed toward cases of higher complexity as clinicians may be less likely to report cases in which toxic alcohol toxicity is unlikely or where medical management is considered relatively uncomplicated. Therefore, poison center data should not be interpreted as a representation of the true statewide incidence of toxic alcohol exposures.

The intent of our study design was to demonstrate the utility of a negative toxic alcohol concentration, based on the recommendation or administration of fomepizole, which did not include all reported toxic alcohol exposures. Specifically, we did not include cases managed with only extracorporeal treatments in this study. Furthermore, the available information in ToxSentry can vary depending on the accuracy and completeness of the poison center records, which in turn depends on the individual recording documentation, leading to information and reporting biases. For example, the hospital LOS was estimated based on the patient’s hospital arrival time and the time of the last poison center follow-up. However, patients may have had longer LOS beyond the time of last follow-up, which could not be abstracted based on available poison center information.

We did not have access to electronic health records from individual hospitals to verify the poison center data. Other variables, such as the number of doses of fomepizole administered and the time to first fomepizole dose, may also be subject to error due to a lack of complete information. Additionally, while most hospitals in the MiPDC catchment area had access to rapid toxic alcohol testing, some hospitals did not, which may have led to a measurement bias. Inherent differences between poison center follow-up guidelines may also exist, which can reflect wider practice pattern differences across geographic regions. Additionally, the higher frequency of acidosis, elevated anion gap, elevated osmolal gap, and hemodialysis in FPIN may be a confounding variable contributing to worse medical outcomes and increased hospital LOS.

The baseline differences in these variables between MiPDC and FPIN could reflect underlying differences in patient acuity or severity of illness at presentation. Although we attempted to account for this difference by including the frequency of “increased anion gap” as a variable in our multivariate models, residual confounding is still possible. On the other hand, some of these baseline differences can be partly attributed to variations in practice patterns. For example, clinicians in MiPDC may be less likely to order surrogate lab tests, such as the osmolal gap or anion gap, because they have ready access to quantitative toxic alcohol levels.

## CONCLUSION

Our retrospective cohort study findings suggest that ready access to rapid confirmatory toxic alcohol testing is associated with shorter hospital length of stay, administration of fewer doses of fomepizole, and improved medical outcomes. Clinical severity of ingestions may represent a significant confounding factor in our study. Expansion of confirmatory toxic alcohol testing capabilities warrants investigation due to the potential for significant translational impacts in optimizing acute patient care and outcomes.

## Supplementary Information





## Figures and Tables

**Table 1:** Descriptive variables in a study comparing suspected toxic alcohol exposures between two U.S poison centers: MiPDC (with a 12–24 hour toxic alcohol testing turnaround time) and FPIN (with a 5–7 day turnaround time).

Variable	MiPDC [n = 590]	FPIN [n = 373]	*P* value
Patient Characteristics			
Age (years)^a^	43.0 (28.0, 56.0)	47.0 (35.0, 58.0)	<.001
Intent			<.001
Intentional	353 (75.3)	280 (88.9)	
Unintentional	116 (24.7)	35 (11.1)	
Intentional–suspected suicide	270 (45.8)	210 (56.3)	.001
Positive history of toxic alcohol ingestion**	269 (45.6)	178 (47.7)	.52
Dialysis	123 (20.8)	164 (44.0)	<.001
Dialysis type			<.001
Hemodialysis only	101 (17.1)	125 (33.5)	
Continuous renal replacement therapy only	11(1.9)	22(5.9)	
Hemodialysis and continuous renal replacement therapy performed	11 (1.9)	17 (4.6)	
Neither	467 (79.2)	209 (56.0)	
Other therapies			
Vasopressors	81 (21.7)	62 (16.6)	.02
Intubation	154 (26.1)	115 (30.8)	.01
CPR	17 (2.9)	8 (2.1)	.48
Clinical effects			
Acidosis	306 (51.9)	234 (62.7)	<.001
Increased anion gap	232 (39.3)	189 (50.7)	<.001
Increased osmolal gap	95 (16.1)	189 (50.7)	<.001
CNS depression	194 (32.9)	199 (53.4)	<.001
Confusion	133 (22.5)	72 (19.3)	<.001
Agitation	79 (13.4)	52 (13.9)	.81
Renal failure	53 (9.0)	46 (12.3)	.10
Increased creatinine	156 (26.4)	110 (29.5)	.30
Blurred vision	10 (1.7)	12 (3.2)	.02
Patient outcomes			
Medical outcomes			<.001
No effect	70(12.2)	11(3.0)	
Minor effect	60(10.5)	36(9.9)	
Moderate effect	183 (31.9)	118 (32.5)	
Major effect	222 (38.6)	166 (45.7)	
Death	39 (6.8)	32 (8.8)	
Hospital LOS (hours)^a^	48.0 (20.0, 89.0)	65.0 (41.0, 101.0)	<.001
Doses of 4-MP (count)^a^	1.0 (1.0, 2.0)	2.0 (1.0, 4.0)	<.001
Time until first 4-MP dose^a^	3.0 (1.5, 5.0)	4.0 (2.5, 8.5)	<.001

* MiPDC has a 12–24 hour turnaround time for toxic alcohol testing. FPIN has a 5–7 day turnaround time.

** Patients deemed to have “known toxic alcohol ingestion in their presentation” when ingestion of a toxic alcohol was self-reported by the patient or witnessed by another and subsequently reported to the clinician

Values *n* (%) unless otherwise specified.

a Median (Q1, Q3)

*CNS*, central nervous system; *CPR*, cardiopulmonary resuscitation; *LOS*, length of stay; *4-MP*, 4-methylpyrazole; *MiPDC*, Michigan Poison and Drug Information Center; *FPIN*, Florida’s Poison Information Network.

**Table 2:** Coefficients table for multivariate analysis of hospital length of stay in a study comparing suspected toxic alcohol exposures between two U.S. poison centers: MiPDC (with a 12–24 hour toxic alcohol testing turnaround time) and FPIN (with a 5–7 day turnaround time).

	Coefficients			

Variable	Exp(B)	95% CI for Exp(B)		*P* value
		Lower Bound	Upper Bound	
Poison Center=FPIN (MIPDC reference)	1.183	1.029	1.359	.02
Male (female reference)	1.035	0.909	1.178	.60
Age (years)	1.007	1.003	1.010	<.001
Doses of 4-MP (count)	1.184	1.153	1.216	<.001
Time until first 4-MP dose (hours)	1.045	1.032	1.057	<.001
Increased anion gap	1.550	1.350	1.780	<.001
Intentional (unintentional reference)	1.934	1.638	2.283	<.001

Dependent variable hospital length of stay was log-transformed. Regression coefficients have been exponentiated.

*4-MP*, 4-methylpyrazole; *MiPDC*, Michigan Poison and Drug Information Center; *FPIN*, Florida’s Poison Information Network.

**Table 3:** Parameter estimates of multinominal logistic regression with medical outcomes in a study comparing suspected toxic alcohol exposures between two U.S poison centers: MiPDC (with a 12–24 hour toxic alcohol testing turnaround time) and FPIN (with a 5–7 day turnaround time).

Parameter Estimates					
Medical Outcome ^a^		Odds Ratio	95% Confidence Interval for Odds Ratio		*P* value
			Lower Bound	Upper Bound	
No effect (reference)					
Death					
	Age (years)	1.068	1.025	1.113	<.01
	Doses of fomepizole (count)	1.045	0.735	1.484	.81
	Time until first fomepizole dose (hours)	1.276	1.081	1.507	<.01
	Intentional (unintentional reference)	19.69	2.185	177.465	.01
	Poison Center= FPIN (MIPDC reference)	4.601	0.971	21.808	.06
	Male (female reference)	0.61	0.165	2.257	.46
Major Effect					
	Age (years)	1.04	1.016	1.064	<.001
	Doses of fomepizole (count)	1.261	1.001	1.59	.05
	Time until first fomepizole dose (hours)	1.247	1.068	1.455	.005
	Intentional (unintentional reference)	17.491	6.407	47.756	<.001
	Poison Center= FPIN (MIPDC reference)	4.076	1.251	13.282	.02
	Male (female reference)	0.942	0.409	2.17	.89
Moderate Effect					
	Age (years)	1.028	1.007	1.05	.01
	Doses of fomepizole (count)	1.092	0.867	1.375	.46
	Time until first fomepizole dose (hours)	1.101	0.943	1.285	.22
	Intentional (unintentional reference)	14.362	6.17	33.428	<.001
	Poison Center= FPIN (MIPDC reference)	3.193	0.999	10.205	.05
	Male (female reference)	1.096	0.494	2.433	.82
Minor Effect					
	Age (years)	1.012	0.991	1.034	.27
	Doses of fomepizole (count)	1.224	0.961	1.558	<.001
	Time until first fomepizole dose (hours)	0.895	0.734	1.093	.28
	Intentional (unintentional reference)	1.94	0.817	4.609	.03
	Poison Center= FPIN (MIPDC reference)	4.722	1.408	15.843	.01
	Male (female reference)	1.572	0.641	3.852	.32

*MiPDC*, Michigan Poison and Drug Information Center; *FPIN*, Florida’s Poison Information Network.

**Table 4:** Quantitative concentrations of methanol and ethylene glycol levels in a study comparing suspected toxic alcohol exposures between two U.S, poison centers: MiPDC (with a 12–24 hour toxic alcohol testing turnaround time) and FPIN (with a 5–7 day turnaround time).

Variable	MiPDC *n* = 590	FPIN *n* = 373
Documented methanol concentration available	402 (68.1)	12 (3.2)
Number of positive methanol results*	23 (5.7)	4 (33.3)
Median methanol concentration among positive results	65.5 mg/dL	215.5 mg/dL
Documented ethylene glycol concentration available	419 (71.0)	26 (7.0)
Number of positive ethylene glycol results*	68 (16.2)	13 (50.0)
Median ethylene glycol concentration among positive results	99 mg/dL	70 mg/dL

* A concentration of > 20 mg/dL was deemed a positive methanol or ethylene glycol result.

*mg/dL*, milligrams per deciliter.
