# Supplementary material for: Rapid Turnaround Time for Toxic Alcohol Testing Is Associated with Shorter Hospital Length of Stay
Source: West J Emerg Med. 2026 Jul 16;27(4):1002–10. doi: 10.5811/westjem.56974 (PMC13436647; doi:10.5811/westjem.56974)
Supplement: Supplementary file 1 [file wjem-27-1002-s001.docx]

**Appendix A.** Hospital Length of Stay (LOS) Abstraction Procedures.

**Data Sources**

Hospital LOS data were obtained from examining Poison Center case narratives present in ToxSentry. and poison center case narratives.

**Definition of LOS and Abstraction Process**

Hospital LOS was defined as the duration from documented time of hospital entry to the documented time of discharge or documented time of last poison center follow-up.

Abstraction of hospital LOS has many limitations as poison center records can be vague or incomplete. In order to generate the most accurate estimates for collecting hospital LOS data, we applied the following methodology.

When abstracting the time the patient first arrived at the Emergency Department (ED), use the documented ED entry time whenever available. If a specific time is not provided, the guidelines below were followed and the appropriate code (bolded below) is documented in the “Notes Column” of the Data Abstraction Excel Sheet.

**Codes and Criteria:**

1. **Initial Call ICU - Start Pushed +[#] Hours**

- Apply when there is a clear ED entry time but the initial call came from the ICU. Adjust the start time backward by the specified number of hours.

2. **Initial Call ICU - Start Pushed to Midnight**

- Apply when documentation states general time references like “patient came in last night.”

3. **Initial Call ICU - Start Pushed to Afternoon (18:00)**

- Apply when documentation states general time references such as the patient arrived “yesterday afternoon.”

4. **Initial Call ICU - Start Pushed to Morning (09:00)**

- Apply when documentation states general time references such as the patient arrived “this morning.”

5. **Initial Call ER – Start Pushed +[#] Hours**

- Apply when the initial call came from the ER but an earlier ED entry time is clearly documented. Adjust the start time by the specified number of hours.

6. **Earlier Labs - Start Pushed +[#] Hours**

- Apply when earlier lab results from this patient’s visit exist prior to the first call. Adjust the start time backward by the specified number of hours.

7. **Initial Call ICU – Unknown Hospital Entry Time**

- Apply when the initial call originated from the ICU but no specific ED entry time is documented.
- In these cases, the time of the first call to the poison center is used.

If there was a documented discharge time, that time was used for the end point of a hospital LOS calculation. If not, the time of the last poison center follow up call was used as an estimate for the end point.

**Appendix B.** Doses of Fomepizole Abstraction Procedures.

**Data Sources:** Data related to fomepizole dosing were abstracted from Poison Center case narratives present in ToxSentry.

**Definition of Doses and Abstraction Process:** Fomepizole dosing was defined as the number of discrete doses administered during a patient's hospital stay. The number of individual doses was collected rather than the number of vials of fomepizole as the latter can change depending on the patient’s weight. Abstraction of the number of doses of fomepizole administered can be challenging due to vague or incomplete documentation. In cases in which there was uncertainty, abstractors used the following guidelines to ensure consistency, with the appropriate code (bolded below) documented in the “Notes Column” of the Data Abstraction Excel Sheet.

**Codes and Criteria:**

1. **4MP was recommended but otherwise** **no indication in notes of when it was given or timing**

- Apply when fomepizole was recommended, but there is no indication in the notes of when it was given or the timing AND there is a low clinical suspicion for toxic alcohol poisoning and/or the patient’s status is improving (including labs: anion gap/osmolal gap).
- If fomepizole was coded as “Performed: Yes” under the Therapies section, abstractors can assume that 1 dose of fomepizole was given.
- If fomepizole was coded as “Performed: No” under the Therapies section, abstractors can assume that 0 doses of fomepizole were given.

2. **Clear 4MP loading dose time with low suspicion but no indication of timing or stop time**

- Apply when there is a clear start time for the first dose of fomepizole documented in the case narrative, but no further notes regarding additional doses exist, and there is low clinical suspicion for toxic alcohol poisoning and/or patient status is stable/improving.
- Assume 1 dose of fomepizole was given.

3. **4MP dosing unclear but there is indication and stop and start and timing of doses**

- Apply when there is a documented start time for administration of the first dose of fomepizole, a documented stop time for administration of the last dose of fomepizole, and a specified dosing interval (e.g., Q12h, Q6h), but there is not documentation of individual fomepizole doses throughout that time period, assume fomepizole was given according to the specified interval.

If the case narrative lacks sufficient documentation for the above methods, that chart is to be excluded from statistical analyses involving the number of doses of fomepizole administered.
